# *Caenorhabditis elegans glp-4* Encodes a Valyl Aminoacyl tRNA Synthetase

**DOI:** 10.1534/g3.115.021899

**Published:** 2015-10-10

**Authors:** Suchita Rastogi, Ben Borgo, Nanette Pazdernik, Paul Fox, Elaine R. Mardis, Yuji Kohara, Jim Havranek, Tim Schedl

**Affiliations:** * Department of Genetics, Washington University School of Medicine, St Louis, Missouri 63110; † National Institute of Genetics, Mishima, 411-8540 Japan

**Keywords:** *glp-4*, valine-tRNA synthetase, *C. elegans*, germline, aging, stress resistance

## Abstract

Germline stem cell proliferation is necessary to populate the germline with sufficient numbers of cells for gametogenesis and for signaling the soma to control organismal properties such as aging. The *Caenorhabditis elegans* gene *glp-4* was identified by the temperature-sensitive allele *bn2* where mutants raised at the restrictive temperature produce adults that are essentially germ cell deficient, containing only a small number of stem cells arrested in the mitotic cycle but otherwise have a morphologically normal soma. We determined that *glp-4* encodes a valyl aminoacyl transfer RNA synthetase (VARS-2) and that the probable null phenotype is early larval lethality. Phenotypic analysis indicates *glp-4(bn2ts)* is partial loss of function in the soma. Structural modeling suggests that *bn2* Gly296Asp results in partial loss of function by a novel mechanism: aspartate 296 in the editing pocket induces inappropriate deacylation of correctly charged Val-tRNA*^val^*. Intragenic suppressor mutations are predicted to displace aspartate 296 so that it is less able to catalyze inappropriate deacylation. Thus *glp-4(bn2ts)* likely causes reduced protein translation due to decreased levels of Val-tRNA*^val^*. The germline, as a reproductive preservation mechanism during unfavorable conditions, signals the soma for organismal aging, stress and pathogen resistance. *glp-4(bn2ts)* mutants are widely used to generate germline deficient mutants for organismal studies, under the assumption that the soma is unaffected. As reduced translation has also been demonstrated to alter organismal properties, it is unclear whether changes in aging, stress resistance, etc. observed in *glp-4(bn2ts)* mutants are the result of germline deficiency or reduced translation.

Germline stem cells produce daughter cells that differentiate into gametes. The generation of a sufficiently large number of gametes for reproductive success requires significant germline stem cell activity. The *Caenorhabditis elegans* hermaphrodite exhibits this extensive stem cell activity. At hatching, each hermaphrodite contains two germ cells that proliferate to more than 800 germ cells per gonad arm in the young adult and that continue to proliferate during the subsequent progeny production period (Hubbard and Greenstein 2005; Pazdernik and Schedl 2013). To identify genes that specify the germline stem cell fate as well as genes that are necessary for stem cell proliferation and cell-cycle progression, genetic screens have been conducted in *C. elegans* for mutations that result in an abnormal Germ Line Proliferation (Glp) phenotype. Two genes identified in these screens are of particular interest, *glp-1* and *glp-4*. Mutations in the *glp-1* gene were used to identify the Notch pathway as the key signaling system between the somatic niche and germ cells for the specification of the germline stem cell fate (Austin and Kimble 1987; 1989; Yochem and Greenwald 1989). In *glp-1* loss of function mutants, germline stem cells prematurely enter meiosis to form gametes. Temperature-sensitive (ts) *glp-1* alleles have been identified and used to further understand the role of GLP-1 signaling in the proliferation *vs.* meiosis decision at the cellular level and to identify other genes involved in the stem cell fate decision through suppressor and enhancer screens (*e.g.*, Austin and Kimble 1987; Fox and Schedl 2015; Maine and Kimble 1989). The *glp-4* gene was identified by the ts allele *bn2*; when homozygous mutant embryos or L1 larvae are grown at the restrictive temperature (25°) adults contain only approximately 12 germ cells, which appear to be arrested in prophase of the mitotic cell cycle (Beanan and Strome 1992). When *glp-4(bn2*ts*)* mutant adults are shifted back to the permissive temperature, cell-cycle arrest is reversed and extensive proliferation occurs, restoring the normal pattern of meiotic development and oogenesis. The regeneration of the germline indicates that the arrested cells correspond to the germline stem cell population. *glp-4* was suggested to interact with GLP-1 signaling as mutations reported to be alleles of *glp-4* were isolated in a screen for enhancers of the premature meiotic entry phenotype of *glp-1* partial loss of function (Qiao *et al.* 1995). *glp-1(ts)* and *glp-4(bn2*ts*)* adult mutants grown throughout larval development at the restrictive temperature have 1% or less the normal number of germ cells but have a soma that is morphologically indistinguishable from wild type at the level of light microscopy (Austin and Kimble 1987; Kodoyianni *et al.* 1992; Beanan and Strome 1992), suggesting that only the germline is affected in the mutants. These properties have led *glp-4(bn2*ts*)* and *glp-1(ts)* mutants to be widely used to generate populations of germline deficient adult hermaphrodites for molecular biology experiments to identify genes whose RNAs and proteins are enriched in the germline (*e.g.*, Rosenquist and Kimble 1988; reviewed in Reinke 2006).

Germline proliferation in *C. elegans* has been linked to rapid organismal aging. Elimination of all germ cells by laser ablation results in lifespan extension (Hsin and Kenyon 1999), which appears to be unrelated to the production of oocytes or sperm, whereas mutants that result in germ cell overproliferation cause lifespan shortening (Arantes-Oliveira *et al.*, 2002). Stimulated by the finding that loss of germ cells leads to lifespan extension, researchers have investigated the role of germ cells in signaling for regulation of other organismal characteristics. However, removal of the germline by laser ablation is technically challenging and very low throughput. Therefore, researchers have turned to the use of *glp-1(ts)* and *glp-4(bn2*ts*)* mutants and temperature shifts to generate populations of adult hermaphrodites that have very few germ cells. For example, *glp-4(bn2*ts*)* mutants, as well as *glp-1(ts)* mutants, have been used to examine the role of the germline in aging, stress resistance, pathogen resistance and fat metabolism (*e.g.*, Wang *et al.* 2008; TeKippe and Aballay 2010; Greer *et al.* 2010; Labbadia and Morimoto 2015). Nevertheless, interpretation of findings after use of *glp-4* and *glp-1* mutants to assess the role of germ cell proliferation on organismal properties relies on the assumption that the mutant conditions do not also affect somatic tissues in a way that could influence aging, stress resistance, pathogen resistance and metabolism. This assumption has not been fully investigated.

The molecular identity and null phenotype of *glp-4* are unknown. This limits our understanding of its role in germ cell proliferation, potential interaction with GLP-1 signaling, and interpreting results when *glp-4(bn2*ts*)* is used to generate germ cell−deficient adults for studies of organismal properties such as aging and stress resistance. We used whole-genome sequencing to demonstrate that *glp-4* encodes the valyl aminoacyl transfer RNA (tRNA) synthetase VARS-2 and determined that the null phenotype is early larval lethality, consistent with a major disruption of protein synthesis. Based on phenotypic analysis we find that *glp-4(bn2*ts*)* is partially deficient for valine-tRNA synthetase function in the soma at the restrictive temperature, thus raising the question whether changes in organismal properties in *glp-4(bn2*ts*)* mutants are due to germ cell deficiency or reduced protein synthesis.

## Material and Methods

### *C. elegans* strains

All strains were cultivated on nematode growth medium plates seeded with *E. coli* strain OP50 (Brenner 1974) and were maintained at 20° unless indicated. The strains used in these studies are as follows: N2 (Bristol) as wild type. *glp-4(bn2*ts*)* (grown at 15°), *glp-4(bn2bn39)*, and *glp-4(bn2bn40)* were obtained from Susan Strome. Enhancer of *glp-1(bn18)*, *om14*, was obtained from Eleanor Maine. Y87G2A.5 *(tm3947)* was obtained from the Japanese National Bioresource Project and balanced over *hIn1[unc-54]*, *agef-1(ok1736)/hIn1[unc-101(sy241)]* and F22G12.5(*ok2367*) were obtained from the *Caenorhabditis* Genetics Center. *unc-75(e950) glp-4(bn2*ts*)* and *unc-75(e950) unc-101(m1)* were constructed by standard methods. Phenotypes were scored using a dissecting microscope or Nomarski DIC microscopy with a 40 or 63× objective.

### Molecular methods

Genomic DNA was isolated by a minor modification of the CTAB method of Mello and Fire (1995). Whole-genome sequencing of *glp-4(bn2*ts*)* was obtained by paired-end reads (average 30× coverage) using an Illumina Genome Analyzer through The McDonnell Genome Institute, Washington University, St Louis MO. Following read pair alignment to the *C. elegans* reference genome with BWA (Li and Durbin 2009), five coding sequence changes in a previously mapped genetic interval were identified in *glp-4(bn2*ts*)* that differed from the N2 wild-type reference sequence (WormBase.org, release WS248): C54C8.5 (*glct-5*), nt position 12454271, G->A, Trp->Stop; F22G12.5, nt position 13168458, G->C, His->Asp; Y87G2A.5 (formerly called *vars-2*, see below), nt position 13556729, C->T, Gly->Asp; Y6B3A.1a (*agef-1*), nt position 13616715, C->T, Asp->Asn; and Y71A12B.17 (*gadr-5*), nt position 13985048, A->G, Lys->Glu. To identify the molecular lesion in the *glp-4(bn2*ts*)* intragenic revertants, we isolated total RNA from each mutant strain. Reverse-transcription polymerase chain reaction with three sets of overlapping primers for Y87G2A.5 was then used to generate the corresponding coding region cDNA, which was Sanger sequenced. Feeding RNA interference (RNAi) was performed (Lee *et al.* 2007) for each of the five candidate genes with the use of clones obtained from the Ahringer library (Rual *et al.* 2004), with the *rrf-1(pk1417)* background to largely limit knockdown to the germline (Kumsta and Hansen 2012). RNA *in situ* hybridization was performed as described (Motohashi *et al.* 2006).

### Genetics methods

#### Complementation testing:

We tested deletion alleles for three of the five candidate genes by placing them in *trans* to *unc-75(e950) glp-4(bn2*ts*)*, verifying the genotype at 15°, and then shifting to 25° to examine the phenotype. Only in the case of Y87G2A.5 *(tm3947)*/ *unc-75(e950) glp-4(bn2*ts*)* were abnormal phenotypes observed.

#### Isolation of suppressors of *glp-4(bn2ts)*:

We isolated recessive suppressors of *glp-4(bn2ts)* with an ethyl methanesulfonate mutagenesis screen similar to the method described by Beanan and Strome (1992), except that the screen for fertility was at 24°. We assessed linkage of the suppressor mutation to *glp-4(bn2*ts*)* by constructing *unc-75(e950) glp-4(bn2*ts*)/ glp-4(bn2*ts*)*; *suppressor/+* strains and screening for *unc-75(e950) glp-4(bn2*ts*)* animals that were fertile at 24° in the following generation. We recovered 26 suppressors from ∼1.1 × 10^6^ F2 generation worms, which included suppressors that were both linked and unlinked to *glp-4(bn2*ts*)*. Linked suppressor *bn2oz283* was examined further.

#### Analysis of *glp-1* enhancer *om14*:

A screen for extragenic enhancers of *glp-1(bn18)* was performed previously in an effort to identify new genes involved in GLP-1 Notch signaling (Qiao *et al.* 1995); among the mutations identified was *om14*, which was reported to be an allele of *glp-4*. We used complementation and linkage analysis to test whether *om14* was an allele of *glp-4*. First, *om14/ unc-75(e950) glp-4(bn2*ts*)* animals were generated at 15° and shifted to 25°. We found that at 25° heterozygotes were fertile and segregated ∼1/4 of progeny that displayed the Unc-75 Glp-4 phenotypes, ∼1/4 of progeny that displayed the oogenesis defective phenotype of *om14* (Qiao *et al.* 1995), and ∼1/2 of progeny that were nonUnc fertile hermaphrodites. Second, *om14* was placed in *trans* to *unc-75(e950) unc-101(m1)*, markers that are tightly linked to *glp-4*. In the next generation we found that ∼1/4 of the Unc animals [*unc-75(e950) unc-101(m1)*] displayed the oogenesis defective phenotype of *om14*. Based on finding that *om14* and *glp-4(bn2*ts*)* complement and that *om14* is unlinked to the right arm of chromosome I, *om14* appears not to be an allele of *glp-4*.

### Comparative structural modeling of *glp-4* valine-tRNA synthetase

#### Construction of the model:

The 2.9 angstrom crystal structure of the valine tRNA-synthetase (valRS) from *Thermus thermophilius* [PDB id: 1IVS (Fukai *et al.* 2003)] was used as a template for constructing a structural model of the valRS from *C. elegans* (*glp-4* VARS-2, see below). Protein sequences for valRS and *glp-4* VARS-2 were aligned using ClustalW (Larkin *et al.* 2007). Three general issues were addressed to generate a homology model: 1) substitution of amino acids at aligned positions; 2) deleted regions; and 3) inserted regions.

To address the first issue, we used RosettaDesign (Leaver-Fay *et al.* 2011; Kaufmann *et al.* 2010) to computationally substitute all nonidentical, aligned residues on the valRS structural template to their identity in *glp-4* VARS-2. The repulsive term in the Rosetta full-atom scoring function was drastically reduced, and the amino acids at altered positions were mutated *in silico* to adopt the *glp-4* VARS-2 sequence. Next, several rounds of rotational isomer (rotamer) sampling (Bower *et al.* 1997) were used to resolve steric clashes whereas the repulsive component of the Lennard-Jones term was gradually increased to ∼60% of its standard value (Kaufmann *et al.* 2010). Finally, once all substitutions had been made and clashes resolved, gradient minimization (Payne *et al.* 1992) was performed on each domain in several iterations. With each iteration, the repulsive term was slowly increased to its standard value to alleviate remaining clashes. Although some significant clashes remained, they were outside the regions of interest for this study, and no further effort was made to remove them.

Second, for elements of valRS that are not present in *glp-4* VARS-2, we manually deleted residues from the PDB file, leaving “gaps” in the structure. One side of the gap was manually chosen as the stationary partner (or target to which the other end of the gap would be aligned) whereas the other side was considered the mobile partner. Selection of the stationary and mobile partners was done depending on which end appeared less likely to be hindered by the surrounding structure. A single amino acid was added onto the mobile partner that overlapped with the targeted stationary partner. Harmonic constraints that forced the overlap residue (on the mobile partner) and the template residue (on the stationary partner) to be superimposed were then enforced, and the structure minimized in several steps with gradually increasing repulsive energy. This allowed efficient closure of the deletion gaps.

Third, for inserted stretches of amino acids that were present in *glp-4* VARS-2 but not in valRS, we added them within Rosetta by a well-developed process called fragment assembly (Misura *et al.* 2006). Briefly, fragment assembly employs a user-generated library of 3 or 9 amino acid peptide structures extracted from the protein databank. These fragments are selected to be of similar sequence as the insertion region. A Monte Carlo sampling algorithm selects the combination of short fragments that both optimize the Rosetta fullatom scoring function and satisfy the starting and ending points for the insertion in the structural model. The fragment insertion that results in the best overall score is retained and manually inspected for the best fit, which in this case was the lowest root-mean square deviation (RMSD) compared with the template. RMSD is a cumulative measure of the mean distance between equivalent atoms in the model and template, which is routinely used to compare the similarity of two regions of homologous protein structure.

Next, we positioned the valyl-adenylate substrate in the editing site by structurally aligning a valyl-adenylate from PDB 1IVS onto to the 5′-*O*-(N-(L-threonyl)-sulfamoyl) adenosine substrate in PDB 1WK9 (editing domain only) (Fukunaga and Yokoyama 2005). PDB 1WK9 was then aligned to the model and the coordinates for the valyl-adenylate substrate were transferred to the model.

Finally, once the model amino acids were fully substituted, insertions/deletions integrated, and the valyl-adenylate substrate added, full structural refinement (Misura *et al.* 2006) using constraints on the template yielded the completed model (see Figure 4). All structural manipulations and calculations were performed using standard and custom Rosetta programs (Leaver-Fay *et al.* 2011; Kaufmann *et al.* 2010) and all visualization performed using PyMol (DeLano 2002; The Pymol Molecular Graphics System, version 1.3. http://pymol.org/).

#### Modeling mutations and paralogous substitutions:

Amino acid changes were modeled using RosettaDesign. Briefly, we performed Monte Carlo sampling of discrete rotamer side-chain conformations on a fixed backbone. This is referred to as “fixed-backbone” design and involves computationally cycling through predefined, low-energy conformations of a particular amino acid, calculating the whole structure energy for each conformation and finding the one with the lowest energy (Leaver-Fay *et al.* 2011; Kaufmann *et al.* 2010). After fixed backbone mutation/substitution, a number of small perturbations in the backbone and side-chain torsion angles are applied in combination with gradient minimization. This allows the backbone to be repositioned along an energy gradient based on new requirements from changing an amino acid identity. In each step, both the tRNA and the bound valyl-adenylate substrate were constrained to their original position (such that they could move, but such motion was heavily penalized by the scoring function). We performed flexible backbone optimization iteratively until no further improvement in energy due to the sequence change was observed (Misura *et al.* 2006).

## Results

### Molecular Identity of the *glp-4* locus

**Figure 1:**
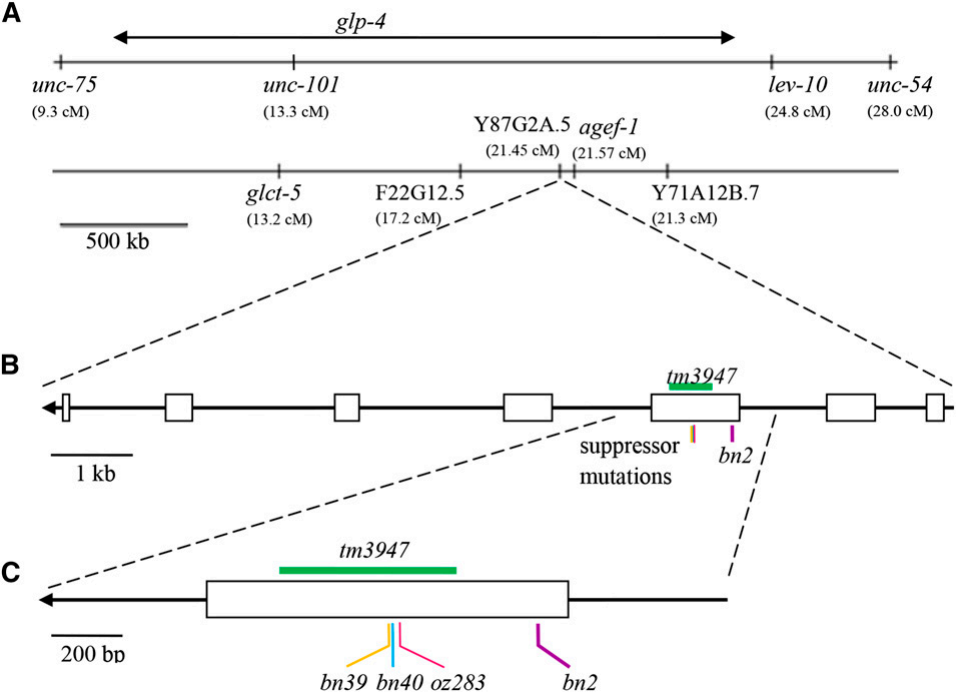
Relative positions of genetic loci and mutant alleles within the genetic and physical map regions around *glp-4*. (A) Displays the genetic map on the right arm of Chromosome I with morphologic loci in the region used to localize *glp-4* indicated (top) and the physical map (bottom) of the region with genes that contain missense mutations from whole genome sequencing indicated. (B) Shows the intron–exon structure of Y87G2A.5, which encodes the cytoplasmic valyl aminoacyl transfer RNA synthetase VARS-2. (C) Shows an expansion of exon 3 with the relative position of *glp-4* genetic lesions characterized in this paper indicated. Arrows in (B) and (C) are included to indicate the direction of transcription, with 3′ to the left. Diagrams are drawn to scale based on nucleotide position on Chromosome *I*. The *tm3947* deletion spans nucleotide positions 13,555,971 to 13,556,472. The *bn2*, *bn39*, *bn40* and *oz283* point mutations occur at positions 13,556,729, 13,556,285, 13,556,288 and 13,556,310, respectively.

The *glp-4* locus was previously mapped genetically to an approximately two-megabase interval between markers *unc-75* and *lev-10* on Chromosome I (Beanan and Strome 1992). We performed whole-genome sequencing (Hobert 2010) of the *glp-4(bn2*ts*)* reference allele-containing strain and identified five candidate genes in the mapped region that contained coding sequence changes compared to the Bristol N2 wild-type reference genome (see the section *Materials and Methods*; Figure 1). To determine which of the five genes encodes *glp-4*, we performed RNAi knockdown of each in the *rrf-1* mutant background, which is proficient for RNAi in the germline but largely defective for RNAi in the soma (Kumsta and Hansen 2012). We then assessed the treated animals at 20° for a sterility phenotype similar to that observed for *glp-4(bn2*ts*)* at 25°. Only knockdown of Y87G2A.5 displayed a sterile phenotype, showing a strong reduction in germ cell number and little or no gametogenesis. We next performed complementation tests for the three candidate genes in the mapped region where deletion alleles were available from the knockout consortium (C. elegans Deletion Mutant Consortium 2012): Y87G2A.5 *(tm3947)*, *agef-1(ok1736)* [Y6B3A.1], and F22G12.5 (*ok2367*). Only Y87G2A.5 *(tm3947)* failed to complement *glp-4(bn2*ts*)*. The *trans*-heterozygotes, although weakly fertile at 15°, are fully sterile at 25° with a limited number of germ cells (also see below). Three tightly linked, possibly intragenic, suppressors of the *glp-4(bn2*ts*)* temperature sensitive sterility have been identified, *bn2bn39*, *bn2bn40* and *bn2oz283* (Beanan and Strome 1992; see the section *Materials and Methods*). The coding sequence of Y87G2A.5 in each of the suppressors was sequenced; in addition to the original *bn2* missense change in exon 3, we found that the suppressors contained a second missense mutation in exon 3 (Figure 1). These findings together provide strong evidence that *glp-4*, defined by allele *bn2*, encodes a valyl aminoacyl tRNA synthetase (VARS-2, Y87G2A.5). Based on the large body of literature on *glp-4*, the gene name will remain *glp-4*, while the protein will be identified as VARS-2 to indicate its molecular identity.

**Figure 2:**
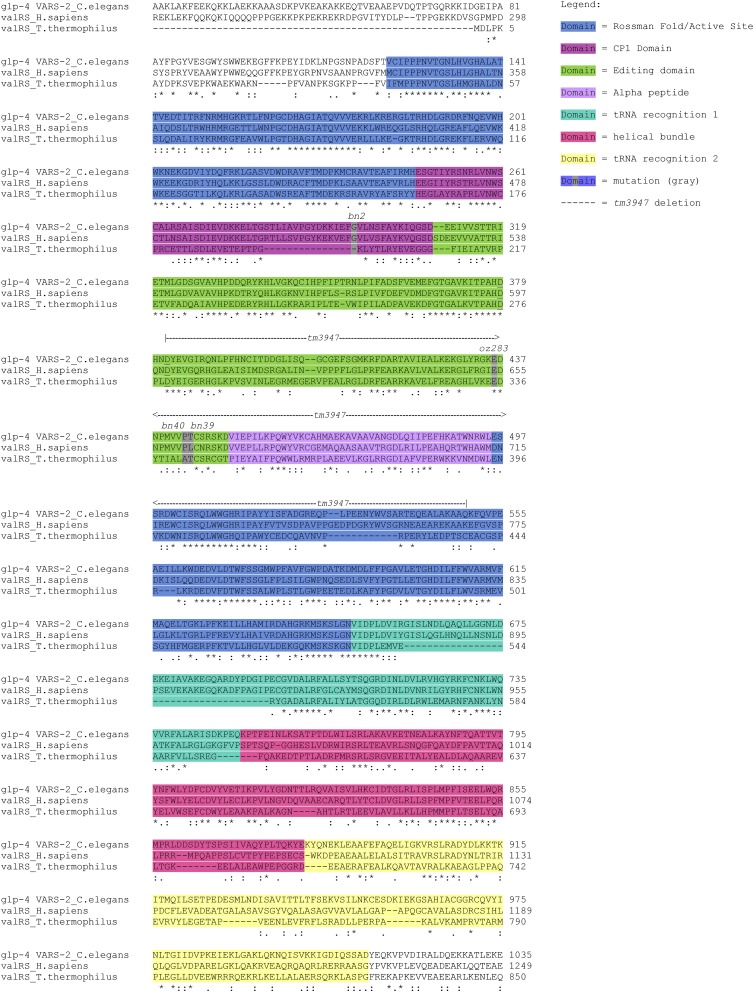
Alignment of *C. elegans glp-4* VARS-2 with human and *Thermus thermophilus* orthologs. Alignment performed with ClustalW (Larkin *et al.* 2007). Protein domains are color coded—the split Rossman fold in blue, CP1 domain in magenta, editing domain in green, alpha peptide in purple, transfer RNA (tRNA) recognition domain 1 in turquoise, helical bundle in pink, and tRNA recognition domain in yellow; this color code is used again in Figures 4A to indicate the relevant domains in the 3D structures. Point mutations (*bn2*, *oz283*, *bn40*, and *bn39*) are marked in the alignment in gray, whereas the deletion allele *tm3947* is indicated by the dashed line above the alignment. The editing active site aspartates D379 and D382 are underlined. Below the alignment, residues that are identical (*), strongly similar (:), or weakly similar (.) for all three proteins are indicated. Note that *glp-4* VARS-2 and human valRS are more closely related to each other than to *T. thermophilus*.

*glp-4* VARS-2 is a cytoplasmic class I valyl aminoacyl tRNA synthetase that catalyzes the transfer of valine to its cognate tRNA for protein synthesis. *glp-4* VARS-2 has two tRNA recognition domains (1 and 2); a split class 1 Rossmann-fold, which functions in catalyzing the synthesis of aminoacyl-adenylate and aminoacyl-tRNA^val^; an editing domain; and the connective polypeptide (CP1) domain, which also functions in post-transfer editing (Figure 2, Fukai *et al.* 2000). *tm3947* deletes 502 base pairs and inserts four base pairs, removing part of the editing domain, all of the CP1 and alpha peptide domains and part of the Rossmann-fold. Thus *tm3947* is likely a null allele. *bn2* is a missense mutation, G296D, in the CP1 domain. Intragenic revertants *bn2bn39*-T444M, *bn2bn40*-P443L, and *bn2oz283*-E436K, are missense mutations in the adjacent editing domain (Figure 2; also see *Structural analysis of glp-4 VARS-2*).

### *glp-4* VARS-2 has somatic functions

**Figure 3:**
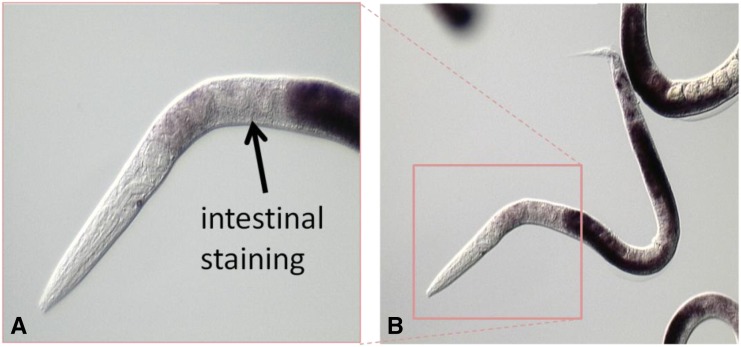
*In situ* hybridization demonstrates expression of *glp-4* VARS-2 in the germline and soma. Higher (A) and lower (B) magnification views of a young adult hermaphrodite showing accumulation of *glp-4* mRNA by *in situ* hybridization using the method of Motohashi *et al.* 2006. Very strong germline expression (overexposed dark staining) is observed in the two U-shaped gonad arms (B). The anterior region, boxed in (B), is enlarged in (A) to illustrate expression of *glp-4* mRNA in the intestine (dark staining, indicated with black arrow).

The soma of *glp-4(bn2*ts*)* homozygotes grown at the restrictive temperature throughout development does not have any obvious morphological abnormalities by light microscopy (Beanan and Strome 1992). Therefore, it was of interest whether *glp-4* VARS-2 has somatic functions. *tm3947*-null homozygotes arrest during early larval development. Although there is some variability in animal size, based on somatic gonad and germ cell counts (from 4–12 cells) the arrest is in the L1 stage. By contrast, *tm3947* heterozygotes are morphologically normal. These results are consistent with slow growth and larval lethality observed in genome-wide RNAi screens with Y87G2A.5 (Simmer *et al.* 2003; Nehme *et al.* 2010; Dunbar *et al.* 2012). *In situ* hybridization reveals that *glp-4* mRNA is expressed in the intestine and somatic gonad, in addition to strong expression in the germline (Figure 3). Thus *glp-4* VARS-2 is expressed and functions in both the germline and soma.

Based on the germline phenotype, *bn2* is a partial loss of function mutation; *bn2* fails to complement the *tm3947* deletion allele for the reduced germ cell number phenotype at 25° and the reduced germ cell number can be phenocopied by RNAi of *glp*-4 VARS-2. In addition to defects in germline development, *glp-4(bn2*ts*)* is also partially defective in somatic function. *bn2/tm3947 trans*-heterozygotes at 25° are slow growing, reaching adulthood more than a day later than the *tm3947/+* control, and have a protruding vulva that often results in bursting in adults. Thus, notwithstanding the apparently normal somatic morphology of *bn2* homozygotes, *glp-4* VARS-2 activity is partially compromised in the soma of *glp-4(bn2*ts*)* animals at 25°.

### Structural analysis of *glp-4* VARS-2

#### Structural homology model:

**Figure 4:**
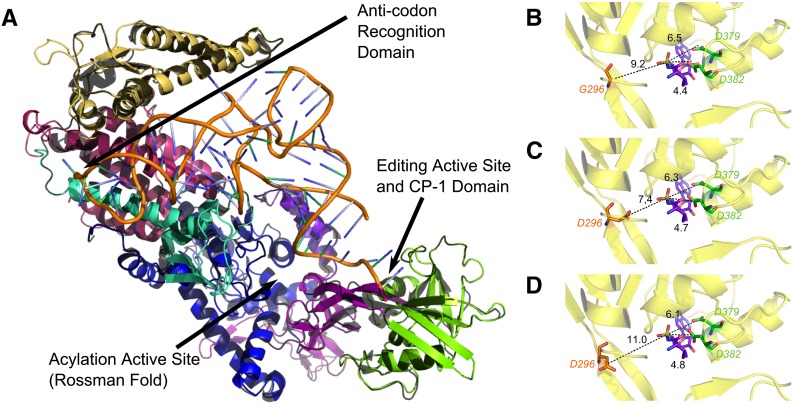
Homology model of the three-dimensional structure of *glp-4* VARS-2 from *C. elegans* (solid, multicolored as described in Figure 2) generated using the crystal structure of the orthologous valyl aminoacyl tRNA synthetase valRS from *T. thermophilus* as the template (black). (A) The entire homology model of *glp-4* VARS-2 with conserved domains color coded and regions of interest indicated with arrows: editing active site (CP1 domain in purple and editing domain in green), anti-codon recognition pocket (red) (see Figure 5), the amino acylation active site and tRNA^val^ (orange). (B) Close-up view of the editing pocket with active site residues D379 and D382 (green), peptide backbone (yellow), valyl adenylate (purple) located just right of center in the pocket, wild type G296 on the opposite side of the pocket (red) and distances between aspartate carboxyl or glycine alpha-carbon and the scissile bond (dashed black line) indicated. (C) Close-up view of the editing pocket in *bn2* G296D where the carboxyl of the substituted aspartate 296 is 7.4 angstroms from the scissile bond and is proposed to catalyze inappropriate deacyalation of correctly charged Val-tRNA^val^. (D) Close-up view of the editing pocket in *bn2bn40* intragenic suppressor, where *bn40* P443L (outside of the close-up region) results in displacement of *bn2* substituted aspartate so that it is now 11 angstroms from the scissile bond.

We have used structural analysis to provide insight into (a) the effect of *bn2* on *glp-4* VARS-2 activity; (b) the mechanism by which the intragenic revertants suppress the effect of *bn2*; and (c) the unique function of *glp-4* VARS-2 relative to the valyl-tRNA synthetase paralog VARS-1. The crystal structure of the *T. thermophilus* valRS has been determined (Fukai *et al.* 2000; 2003). Each of the structural domains identified in valRS is readily discerned in the *glp-4* VARS-2 sequence (Figure 2) and secondary structure predicted with DSSP (Kabsch and Sander 1983). Although the overall primary sequence identity is modest (37%), the aligned predicted secondary structure of the two proteins in placement of helical and sheet segments is in 92% agreement. These findings, along with previous work that successfully generated a homology model with proteins of similar modest sequence identity (Misura *et al.* 2006), indicate that a reasonably accurate structural homology model for *glp-4* VARS-2 can be generated using valRS as a template. An initial model was generated as described in the *Materials and Methods* section and assessed by visual examination of the model to look for obvious structural inaccuracies and by computation of the model RMSD, which is a quantitative scalar measure of the generated model backbone’s fit relative to the template backbone. Our structural model for *glp-4* VARS-2 (Figure 4A) has a backbone RMSD of 0.8 angstroms (determined by pyMol alignment) from the starting template, indicating that the crystal structure can be used for homology modeling of the *C. elegans* protein with only modest backbone rearrangement.

#### Effect of *bn2* on GLP-4 VARS-2 activity:

During the process of charging tRNAs with amino acids for subsequent translation, an amino acid residue can be misacylated to result in a noncognate tRNA-amino acid pair. Class I tRNA synthetases can correct such errors via post-transfer editing, *i.e.*, the removal of an amino acid that is attached to a noncognate tRNA. This is an essential part of the “double-sieve” mechanism critical for maintaining translational fidelity. Isosteric threonine charged onto tRNA^val^ is the major misacylation that is post-transfer edited by valyl-tRNA synthetases. Although a number of residues have been shown to be critical for coordination and recognition of the amino-acylated tRNA during post-transfer editing, hydrolytic cleavage of the misacylated Thr-tRNA^val^ substrate is thought to take place in a hydrophilic pocket of the tRNA’s editing domain. This domain contains a pair of conserved aspartates (D379 and D382, in green in Figure 2 and Figure 4) that are about 6.5 and 4.4 angstroms from the scissile bond (Fukai *et al.* 2000; Fukunaga and Yokoyama 2005). One of these aspartate residues deprotonates an adjacent water molecule to create an activated hydroxyl ion, which nucleophilically hydrolyzes the Thr-adenylate bond of the noncognate amino acid tRNA^val^ substrate.

*glp-4(bn2*ts*)* G296D lies just inside a region of the valyl-tRNA synthetase called the CP1 domain (Figure 2 and Figure 4), a region that links the Rossman-fold of class I tRNA synthetases to the editing domain (Fukai *et al.* 2000), and plays an important editing role of its own for the isoleucyl, leucyl, and valyl-tRNA synthetases (Chen *et al.* 2000; Sarkar *et al.* 2007). In the wild-type model, G296 resides on a flexible region of the CP1 domain, opposite from the catalytic aspartates in the hydrophilic editing pocket. The glycine 296 alpha-carbon sits approximately 9.2 angstroms from the scissile bond (Figure 4B). In the *bn2* G296D mutant, the aspartate 296 carboxyl is now 7.4 angstroms from the scissile bond (Figure 4C). Given this short distance and the fact that the environment is solvent accessible, it is possible that mutant aspartate 296 can act as a proton acceptor to activate a water molecule for nucleophilic attack of the scissile bond. Although the *bn2* aspartate 296 mutation is in a position opposite the native catalytic aspartates, it is oriented such that the acidic side-chain is immediately adjacent to the valyl-adenylate substrate.

We propose that *bn2* aspartate 296 catalyzes inappropriate (rogue) deacylation of the correctly charged Val-tRNA^val^. Catalysis due to such a mechanism would not necessarily require the stringent positioning enforced by the binding pocket in the editing domain that is required for deacylation of mischarged Thr-tRNA^val^ via the native catalytic aspartate residues. In fact, we speculate that the temperature sensitivity exhibited by the *glp-4(bn2*ts*)* mutant is due to an increased tendency to mis-deacylate Val-tRNA^val^ at elevated temperatures. Because the G296D mutation is located in a flexible region opposite the active site residues, lower temperatures may provide insufficient dynamic accessibility of mutant aspartate 296, resulting in low levels of inappropriate deacylation of Val-tRNA^val^. At greater temperatures, catalysis of the inappropriate deacylation reaction would be increased, as aspartate 296 would be brought into contact with the valyl-adenylate substrate more frequently. Thus we propose that *bn2* G296D causes inappropriate deacylation of Val-tRNA^val^ at elevated growth temperatures, leading to reduced levels of correctly charged Val-tRNA^val^ that result in decreased bulk protein synthesis.

The proposed rogue deacylation of correctly charged Val-tRNA^val^ by the *bn2* mutant protein can be considered as antimorphic, where inappropriate activity results in less Val-tRNA^val^ and thus failure to complement the deletion allele *tm3947*. The recessive nature of the *bn2* mutation is likely explained by *glp-4* VARS-2 activity from the wild-type allele catalyzing and releasing sufficient Val-tRNA^val^ for normal function, consistent with *tm3947* being recessive. Other examples of recessive antimorphs have been described, *e.g.*, *sup-10(n983)* and *mett-10(oz36)* (Greenwald and Horvitz 1986; Dorsett *et al.* 2009), although the molecular mechanisms appear to be very different. The prior failure to recover new loss of function alleles in *trans* with *bn2* in a noncomplementation screen (Beanan and Strome 1992) was probably because of severely reduced fertility, as we observed for *tm3947/bn2* at the permissive temperature, which would be exacerbated by a general reduction in progeny production that typically occurs following chemical mutagenesis.

#### Suppression of bn2 G296D by the intragenic revertants:

The *glp-4(bn2bn39)* T444M and *glp-4(bn2bn40)* P443L suppressor mutations lie in the editing domain, approximately 23 and 21 angstroms from the *bn2* G296D mutation, respectively. Each sits in what is likely a ‘hinge’ region that joins the editing and CP1 domains to the rest of the protein. Modeling of these mutations into the G296D model introduces local steric clashes around residues 443 and 444 that, when resolved, alter the orientation of the beta-sheet linker in which they are contained. In the case of *glp-4(bn2bn39)* T444M, minimizing the clashes increases the distance between the *bn2* aspartate 296 mutation and the scissile bond from 7.4 to 8.5 angstroms, as the beta-sheet linker is “pushed” away from the editing domain. This is likely due to the physical change when replacing a relatively polar amino acid (threonine) with a larger, hydrophobic residue (methionine). Specifically, the surrounding region may “collapse” to minimize the solvent exposure of methionine, thus shifting the “upstream” beta-sheet. For *glp-4(bn2bn40)*, the P443L mutation increases the distance between the *bn2* aspartate 296 mutation and the scissile bond from 7.4 to 11 angstroms as well as rotating the carboxyl side-chain away from the valyl-adenylate substrate (Figure 4D). Replacement of constraining proline 443 with the larger side chain in leucine results in a major disruption in the beta-sheet in which it resides. For the third intragenic suppressor *glp-4(bn2oz283)*, E436K, the side chain of lysine 436 faces the external solvent and does not reveal an obvious disruption of the structure and positioning of aspartate 296. Thus the mechanism of suppression by E436K must be more indirect, with the structural model providing little insight. In sum, the intragenic suppressor mutations T444M and P443L result in displacement of the mutant aspartate in the editing pocket through steric effects that are propagated to *bn2* G296D (Figure 4D), such that its ability to catalyze inappropriate deacylation of the cognate Val-tRNA^val^ is reduced.

Intragenic suppressors of *bn2* were isolated at 100- to 1000-fold lower frequency following EMS mutagenesis than typical loss of function frequency, which was interpreted by Beanan and Strome (1992) as the *bn2* mutant not being reverted through simple loss of gene activity and that the gene product is likely to be essential. Both inferences were correct based on our findings: (a) *glp-4* VARS-2 is an essential gene; and (b) only a small subset of intragenic mutations are expected to suppress *bn2*, those that displace G296D in the editing pocket and thus decrease its inappropriate deacylation activity without otherwise damaging tRNA synthetase function. *bn2bn40* was observed to be a stronger intragenic suppressor (weakly dominant, larger brood size) than *bn2bn39* (fully recessive) (Beanan and Strome 1992), which is consistent with the structural model where *bn2bn40* P443L results in a greater displacement of the *bn2* aspartate 296 from the valyl-adenylate substrate, compared with *bn2bn39* T444M, as well as rotating the carboxyl side-chain away from the scissile bond (Figure 4D).

#### Unique function of *glp-4* VARS-2:

**Figure 5:**
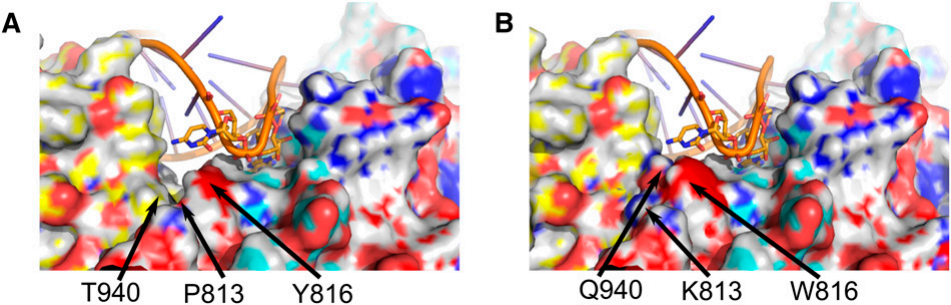
Enlargement of anticodon binding pocket homology model for the two *C. elegans* valRS paralogs *glp-4* VARS-2 (A) and VARS-1 (B). In both panels the tRNA^val^ anticodon is shown as a stick structure interacting with the pocket. (A) The *glp-4* VARS-2 anticodon binding pocket contains tyrosine at residue 816, proline at 813 and threonine at 940, which generate a pocket that is large enough to accommodate anticodons whose wobble positions contain either purines or pyrimidines. (B) The VARS-1 binding pocket contains bulky substitutions at these residues (816 is tryptophan, 813 is lysine and 940 is glutamine) that result in steric clashes when the antiocodon wobble position contains purines, suggesting that VARS-1 is unable to efficiently charge tRNA^val^ for the codons GUC and GUU.

*C. elegans* contains a second valyl-tRNA synthetase paralog, *vars-1*. To determine why *vars-1* is unable to compensate for the reduced ability of *glp-4bn2* and *tm3947* to produce Val-tRNA^val^, we aligned sequences in *glp-4* VARS-2 that are in direct contact with the anticodon with the same region in VARS-1, and then mapped the VARS-1 anti-codon recognition residues onto our model (Figure 4A and Figure 5). The model reveals several bulky substitutions in the immediate vicinity of the anticodon in VARS-1. Tyrosine 816 of *glp-4* VARS-2 lies in direct contact with the wobble position in the anti-codon; VARS-1 has a bulkier tryptophan substitution for residue 816. Two nearby residues in *glp-4* VARS-2, proline 813 and threonine 940, also have substitutions in VARS-1 to bulkier amino acids, lysine and glutamine, respectively. Thus, although the anticodon recognition pocket in *glp-4* VARS-2 appears to easily accommodate binding of either purines or pyrimidines in the wobble position, the three bulky substitutions in VARS-1 decrease the pocket volume, sterically preventing binding anticodons with larger purines in the wobble position. Therefore VARS-1 is not able to efficiently charge tRNA^val^ that recognize valine codons GUC and GUU, which provides an explanation for the unique and essential function of *glp-4* VARS-2 in viability, germ cell proliferation, and protein synthesis.

## Discussion

*glp-4* encodes the VARS-2 valyl aminoacyl tRNA synthetase [Y87G2A.5] based on (a) genetic mapping of the canonical allele *bn2* to a 2 megabase region surrounding Y87G2A.5, (b) finding the G296D missense change in VARS-2, (c) finding that of all the genes within the genetically mapped region containing missense mutations in the *glp-4(bn2*ts*)* strain, only RNAi of Y87G2A.5 phenocopies the *glp-4(bn2*ts*)* mutant, (d) finding that the *tm3947* deletion in Y87G2A.5 fails to complement *glp-4(bn2*ts*)*, and (e) finding that tightly linked *glp-4(bn2*ts*)* suppressor mutations are missense changes within VARS-2. Genetic analysis indicates that *glp-4(bn2*ts*)* is a partial reduction-of-function mutation while structural modeling suggests that *bn2* G296D in the CP1 domain results in substitution of an asparate in the editing pocket opposite to the catalytic aspartates, in a position where it can catalyze inappropriate deacylation of the correctly charged Val-tRNA^val^ and thus reduce levels of Val-tRNA^val^. Structural modeling of the *bn2bn39* T444M and *bn2bn40* P443L intragenic suppressor mutations suggests that the bulkier missense changes result in displacement of the *bn2* asparate 296 in the editing pocket so that it is less able to efficiently catalyze inappropriate deacylation of Val-tRNA^val^. *glp-4* VARS-2 is essential for somatic and germline development as the likely null phenotype is early larval lethality. The widely used allele *bn2*, in addition to its germline defects at the restrictive temperature, is also defective in somatic development and function as *glp-4(bn2*ts*)/glp-4* null displays slow growth, protruding vulva, and bursting at the vulva. These findings reinforce the caution that a loss-of-function mutant that displays a strong morphologic phenotype in one cell type but no morphologic phenotype in other cell types does not preclude that the mutant is also partially defective in other cell types, although not manifested at a morphologic level. A partial loss-of-function allele of the arginyl-tRNA synthetase *rars-1(gc47)* was identified in a forward genetic screen for mutations that result in resistance to hypoxia (Anderson *et al.* 2009). Although *rars-1(gc47)* mutants, like *glp-4(bn2*ts*)* mutants, are morphologically normal, they nevertheless have reduced protein translation rate. Thus by analogy, *glp-4(bn2*ts*)* mutants at the restrictive temperature are likely to be partially defective in protein synthesis, which lead to the observed mutant phenotypes.

*glp-4(bn2*ts*)* and *glp-1(ts)* mutants have been used widely to generate germline deficient adults to study the role of signaling between germ cells and somatic tissues for organismal aging, stress resistance, pathogen resistance, and metabolism. Interpretations of results from such studies rely on the assumption that the *glp-4(bn2*ts*)* and *glp-1(ts)* germ cell−deficient animals have a soma that is unperturbed. However, our results indicate that *glp-4(bn2*ts*)* at the restrictive temperature is partial loss of function in the soma. In addition, a large body of literature in *C. elegans* has implicated partial inhibition of translation in lifespan extension, stress resistance and pathogen resistance (*e.g.*, Hansen *et al.* 2007; Pan *et al.* 2007; Anderson *et al.* 2009; Wang *et al.* 2010; Dunbar *et al.* 2012). *glp-4(bn2*ts*)* mutants, although germline deficient, very likely have partial inhibition of translation due to reduced levels of charged Val-tRNA^val^ . Thus it is unclear whether organismal lifespan extension, stress resistance, pathogen resistance, and increased fat accumulation observed in *glp-4(bn2*ts*)* mutants is a result of germline deficiency, reduced protein synthesis or a combination of both phenotypes. It should be noted that *glp-1* also has functions in the larval/adult soma (Mango *et al.*, 1991; Berry *et al.*, 1997; Singh *et al.*, 2011), which raises concern about interpretation of studies of organismal properties of germline deficient *glp-1(ts)* mutants. We suggest that attribution of organismal phenotypes to signaling from the germline is best demonstrated through laser ablation of the germline precursors, Z2 and Z3 (Kimble and White 1981).

*glp-4(bn2*ts*)* mutants display two intriguing temperature shift phenotypes that can now be correlated with protein translation. Partial disruption of protein translation (at 25°) through decreased levels of charged Val-tRNA^val^ leads to mitotic cell cycle arrest in the stem cell population, whereas restoration of protein translation in the adult (following shift back to 15°) results in release of mitotic cell-cycle arrest, extensive stem cell proliferation, and the resulting reformation of the normal germline distal-proximal polarity of meiotic prophase progression and gametogenesis. These phenotypes are remarkably similar to adult reproductive diapause (Angelo and Van Gilst 2009; Seidel and Kimble 2011) where complete food deprivation at the late L4 stage leads to cell cycle arrest among the stem cells, autophagy/apoptosis mediated resorption of the remaining germline and extended lifespan; upon refeeding there is extensive stem cell proliferation and reformation of the polarized meiotic and gametogenic germline organization. Studies of *glp-4(bn2*ts*)* may thus contribute to understanding of how physiological perturbations (changes in levels of charged Val-tRNA^val^ and/or protein translation) affect entrance and exit from adult reproductive diapause and used to model reformation of the polarized germline organization in adults that contain only a small stem cell population.
